# Sensory Attributes Driving Preference for Wild Rocket (*Diplotaxis tenuifolia*) Leaves Tasted as a Single Ingredient and as a Part of a Recipe

**DOI:** 10.3390/foods13111699

**Published:** 2024-05-28

**Authors:** Antonio Raffo, Irene Baiamonte, Gina Rosalinda De Nicola, Valentina Melini, Elisabetta Moneta, Nicoletta Nardo, Marina Peparaio, Eleonora Saggia Civitelli, Fiorella Sinesio

**Affiliations:** 1 CREA-Research Centre for Food and Nutrition, Via Ardeatina, 546, 00178 Rome, Italy; irene.baiamonte@crea.gov.it (I.B.); valentina.melini@crea.gov.it (V.M.); elisabetta.moneta@crea.gov.it (E.M.); nicoletta.nardo@crea.gov.it (N.N.); marina.peparaio@crea.gov.it (M.P.); eleonora.saggiacivitelli@crea.gov.it (E.S.C.); fiorella.sinesio@crea.gov.it (F.S.); 2 CREA-Research Centre for Vegetable and Ornamental Crops, Via dei Fiori, 8, 51017 Pescia, Italy; ginarosalinda.denicola@crea.gov.it

**Keywords:** arugula, *Brassicaceae*, taste, chemesthesis, trigeminal, odour, isothiocyanates, acceptability, hedonic scaling

## Abstract

Two cultivars of wild rocket (*Diplotaxis tenuifolia*), cv. Denver and Marte, were subjected to chemical determination of flavour-related constituents, sensory descriptive analysis, and measurement of liking by consumer test. Consumers evaluated rocket leaves both as a single ingredient and in a recipe formed by a roll of bresaola with also Grana Padano cheese. Sensory analyses showed that Marte was characterized by a more intense bitterness, hotness, and pungency, which corresponded to a higher total GSL content, mainly due to a higher level of dimeric 4-mercaptobutyl GSL. Five clusters of consumers were identified based on their liking scores. When tasting rocket leaves as a single ingredient, three clusters showed a higher liking for the milder cultivar, one cluster showed an opposite preference, while flavour attributes, such as bitterness and hotness, appeared as the main drivers of liking. Differences in liking were no longer found between the two cultivars when rocket leaves were evaluated in the recipe. Therefore, as rocket leaves are generally consumed as a part of a recipe with other ingredients instead of as a single ingredient, in the assessment of consumer preferences, it should not be neglected the influence of the way in which the product is consumed.

## 1. Introduction

Rocket leaves are appreciated worldwide for the distinctive sensory properties that make them a versatile ingredient in many culinary applications [1]. Two main species are cultivated, *Diplotaxis tenuifolia*, often referred to as “wild rocket”, and *Eruca sativa*, generally referred to as “cultivated” rocket, both belonging to the Brassicaceae family. *Diplotaxis tenuifolia* is the most important species economically due to its preferred agronomic and qualitative features in major producing countries [1,2]. In recent decades, the commercial success of rockets has been primarily linked to the marketing of the packaged and ready-to-eat product rather than to the sale of fresh, unprocessed leaves, and currently, rockets represents one of the main species used in the production of ready-to-eat salads [3].

The peculiar sensory profile of rocket leaves forms the basis of consumer appreciation [4]. Recent investigations have highlighted that sensory attributes of rocket leaves may vary greatly depending on many factors, such as genotype [5,6,7], growing season, cultivation practice [2], processing [3], storage [8], packaging [9] and commercial supply chain [10,11] conditions. Moreover, two recent studies have been dedicated to identifying the sensory attributes primarily responsible for consumer liking. In the first study, seven accessions of *Eruca sativa* were examined, including one used commercially [4], while in the second study, a very large and diverse group of commercial samples of the rocket, almost all belonging to different varieties of the *Diplotaxis tenuifolia* species and mainly grown in Italy under different environmental and cultivation conditions, were analyzed [2]. The main finding of the first study was that hotness was the main attribute on which consumers base their liking of rockets, whereas perceived bitterness did not significantly influence their preferences [4]. In the second study, it was found that rocket leaves with high intensity of both pepperiness, bitterness, and hotness, which were associated with higher levels of specific glucosinolates (GSLs), were, on average, less appreciated by consumers, while leaves with greater sugar content were perceived as sweeter, and this resulted in a higher consumer liking [2]. In both cases, consumer preferences were evaluated in a population sample living in the UK, even though cultural and ethnic diversity was represented within the consumer sample to a certain extent. However, consumer attitudes and preferences for food and, in particular, for fresh fruit and vegetables can vary significantly when assessed in different countries [12]. Cultural differences and related consumption habits and familiarity with certain products, tastes, or flavours affect consumers’ perceptions and preferences and are commonly observed when assessing the acceptability of food products in cross-cultural studies [13]. As an example, the distinctive pungency of radish, which, as in the case of rocket, is associated with its isothiocyanate (ITC) constituents, has been observed to differently affect product liking depending on the nationality of the consumers [14]. Differences in culinary history and culture between Italy and Northern European countries have brought in the past diverse consumption habits and familiarity with rockets; thus, cross-country differences in preferences between Italy and other European countries cannot be ruled out. Nowadays, based on anecdotal information reported by producers, it is believed that Italian consumers prefer rocket leaves characterized by a certain hotness, serrated shape, and proper crispiness. However, specific investigations into the preferences of Italian rocket consumers have not been carried out until now.

Moreover, it is known that rocket is generally consumed, at least in Italy, not as a single-ingredient salad but in multi-ingredient recipes, such as mixed salads, pasta dishes, pizza, and others, thanks to its ability to add a variety of flavours and colour to food preparation. Thus, in the evaluation of consumers’ preference for different rocket cultivars, an important element that should not be underestimated is the influence that the specific way in which a rocket is actually consumed can have on the overall liking judgment. The hypothesis of this study is that the presence of other ingredients in a recipe could have a marked influence on the liking of the rocket when compared to tasting the leaves as a single ingredient and that certain sensory attributes, such as a high hotness and bitterness, which may be found offensive when rocket is tasted as a single ingredient, might simply be unnoticed by consumers when consumed in a food preparation with other ingredients or even turn out to be appreciated sensory features. This would suggest that, while the previous evaluations of consumer liking were performed by tasting rocket leaves as a single ingredient, a more accurate and comprehensive assessment could be obtained by including the assessment also rocket samples as a part of a recipe.

The aims of the present study were to identify which sensory attributes drive the liking of wild rocket (*Diplotaxis tenuifolia*) leaves in a representative sample of Italian consumers and to investigate how the role of these sensory attributes may be influenced by the presence of other recipe ingredients during tasting. These objectives have been pursued by comparing consumer liking and chemical and sensory properties of two wild rocket cultivars selected for their markedly diverse sensory profile. For the study, rocket leaves samples obtained via commercial real-life processing and distribution conditions were used. Consumer liking was evaluated by tasting rockets both as a single ingredient and as a part of a food preparation, formed by a roll of bresaola with also Grana Padano cheese, which is a common appetizer in Italian cuisine. Moreover, information on the purchase and consumption habits of consumers and on their preferences concerning the shape of rocket leaves were also collected by a questionnaire.

## 2. Materials and Methods

### 2.1. Rocket Leaves Samples

Commercial packaged ready-to-eat wild rocket samples were supplied by a farm located in the province of Salerno and producing Rucola della Piana del Sele, which has recently obtained the recognition of the PGI (Protected Geographical Indication) label [15]. The exact locations and names of the farms will not be reported for reasons of commercial sensitivity. Rocket (*Diplotaxis tenuifolia*) leaves from two cultivars, Denver (ISI Sementi S.p.A., Fidenza (Parma), Italy) and Marte (Blumen Group S.p.A., Milano, Italy), were used to produce the commercial samples used for the study. The rocket was grown in a greenhouse and then processed and packaged according to commercial practices required for the production of ready-to-eat Rucola della Piana del Sele PGI. Rocket leaves were harvested as a first cut and processed in June. Immediately after packaging, the product (30 bags, each containing 100 g of leaves for each cultivar) was sent via commercial transportation, under refrigeration, to the CREA-Research Centre for Food and Nutrition, where it was quickly transferred to a refrigerated storage room at 6 °C.

### 2.2. Chemical Determinations

#### 2.2.1. Dry Matter and Soluble Solids Content

A sample of leaves (500 g) collected from 5 bags was used to determine the dry matter content by drying in a laboratory oven (at 70 °C, overnight) and then by drying in a vacuum oven (at 70 °C, overnight), and to obtain a homogenate used for determination of soluble solids content by a digital refractometer (DBR 35).

#### 2.2.2. Sugar Content

Five grams of the fresh rocket leaves homogenate was added with 20 mL of 85% ethanol and mixed by using an Ultra-Turrax for 2 min (at 9200 rpm). After shaking for 1 h at 50 °C, the mixture was centrifuged at 3000× *g* and 20 °C for 15 min, 10 mL of supernatant was taken, dried by rotavapor, and then taken up again with 0.5 mL of acetonitrile and water 50:50, (*v*:*v*). The obtained extract was filtered with a 0.45 µm syringe filter (RC membrane, Goettingen, Germany) and then injected (20 μL) for chromatographic analysis. The HPLC analyses were performed on an HP Series 1100 Agilent system equipped with a refractive index detector; the analytical column was a Luna NH2 (3µ, 4.6 × 150 mm), with Security Guard Cartridge NH2 (4 × 3.0 mm), thermostated at 30 °C. The mobile phase was acetonitrile/water 75:25 (*v*:*v*), with a flow rate of 1 mL/min. Quantification was accomplished on the basis of calibration curves obtained by standard solutions of pure compounds (Merck Life Science S.r.l., Milano, Italy). Triplicate extractions and chromatographic analyses were carried out.

#### 2.2.3. Volatile Organic Compounds (VOCs)

Determination of VOCs was performed by a method based on HeadSpace Solid Phase Microextraction (HS-SPME) for volatile isolation and Gas Chromatography-Mass Spectrometry (GC-MS) for their analytical determination, as described elsewhere [16]. Triplicate HS-SPME extractions and GC-MS analyses were performed on the aqueous mixture obtained from each rocket sample.

#### 2.2.4. Glucosinolates

Glucosinolates (GSLs) were analyzed according to the EU standard procedure ISO 9167:2019 [17] with some modifications. Freeze-dried rocket leaves were reduced to a fine powder. Samples (400 mg) were extracted for 5 min at 80 °C twice with ethanol/water (5 mL, 7:3 *v*/*v*), using a VDI 25 homogenizer (VWR, Darmstadt, Germany) and then centrifuged with a 5810R centrifuge (Eppendorf AG, Hamburg, Germany) at 4000 rpm for 10 min at 10 °C. Supernatants were combined, and each extract (2 mL) was loaded onto a mini-column filled with DEAE-Sephadex A-25 anion-exchange resin (0.6 mL, GE Healthcare, Chicago, IL, USA) conditioned with 25 mM sodium acetate buffer (pH 5.6). After washing with buffer (3 mL), purified sulfatase (200 µL) was loaded onto the mini-column and left for overnight reaction. Extraction and desulfation were performed in duplicate. Desulfo-GSLs (DS-GSLs) were then eluted with ultra-pure H_2_O (3 mL) and analyzed on an HPLC (Shimadzu, Kyoto, Japan) system equipped with a Synergi Fusion-RP column (250 × 4.6 mm, 4 µm particle size), thermostated at 30 °C, and having a PDA detector. The chromatography was performed at a flow rate of 1 mL/min, eluting with a gradient of H_2_O (A) and CH_3_CN (B) following the program: 1 min 1% B; 33 min linear gradient up to 33% B; hold at 33% B for 7 min; 3 min linear gradient down to 1% B. DS-GSLs were detected by absorbance monitoring at 229 nm. Identification of the peaks was performed based on retention times and UV spectra of standards available in our library. The GSL amount was quantified by using a calibration curve of pure DS-sinigrin solution (range from 0.06 to 1.93 mM) and the relative proportionality factor (RPF) of each individual DS-GSL [18]. Pure DS-sinigrin was obtained from commercial sinigrin (analytical standard, Supelco, Darmstadt, Germany). RPF values for quantification were as follows: 1.07 for DS-glucoraphanin, 1.04 for DS-glucoerucin, 1.0 for DS-dimeric 4-mercaptobutyl GSL, 0.28 for DS-4-hydroxyglucobrassicin and 0.29 for DS-glucobrassicin.

### 2.3. Sensory Analysis

The two rocket cultivars were presented to a panel of experts in a monadic way, coded with three-digit random numbers. Three repeated evaluations of each cultivar were conducted over three sessions on the same day. The evaluation order of the two cultivars was balanced between assessors and sessions to correct for the carry-over effect.

All the panellists had undergone training in accordance with ISO standards for sensory analysis [19] (ISO 8586:2023) and are monitored for their performance [20] (ISO 11132:2021). They had over 20 years of experience in sensory analysis. They had received previous training in rocket leaves evaluation, had developed a specific lexicon for sensory profiling of rockets and gained experience in the sensory evaluation of rockets in the previous two years.

In preliminary sessions for this study, they selected 18 attributes from a previously developed list of attributes [3]. An additional appearance attribute, named leaf shape, was included in the list used for the present study: the extreme points of the scale corresponded to a round-shaped leaf (=0) and to a serrated-shaped leaf profile (=9). Evaluation of appearance attributes was performed on approximately 60 g of leaves for each sample under white light. To measure the intensity of each sensory attribute, a 150 mm unstructured linear scale was used, anchored from 0 to 9 at the extreme points of the scale, corresponding to the lowest and the highest intensity of the attribute. Individual portions of approximately 20 g of leaves were served in plastic plates, marked with a 3-digit random number, and used for odour, flavour, and texture assessment. Specific instructions were provided for the evaluation of odours and off-odours: panellists were instructed to first smell the intact leaves and then to smell them again after breaking some leaves by hand. Then, they tasted the product for measurement of flavour and texture attributes. A mandatory minimum one-minute break was enforced, and water was served to clean the palate between each sample. Evaluations were carried out within individual booths in the sensory lab of CREA under controlled climate conditions in June 2023. Data were acquired using the FIZZ software (v. 2.50; Biosystèmes, Couternon, France).

### 2.4. Consumer Evaluations

#### 2.4.1. Rocket Sample Preparation and Presentation

For the consumer test, the two rocket cultivars, Denver and Marte, were served in two different conditions: (1) as a single ingredient, as evaluated by the sensory panel, and (2) as a part of a recipe (a roll of bresaola, with rocket and Grana Padano cheese). The samples were prepared by rolling a slice of bresaola (about 7 g) stuffed with 6 g of rocket leaves and 1 g of Grana Padano cheese flakes. This roll was the portion for two people, and it was divided into two equal pieces before serving.

#### 2.4.2. Consumers Recruiting and Acceptability Test

A total of 100 respondents were recruited by a service provider in Rome and screened in accordance with specific eligible criteria: be regular consumers of rocket (at least once a month), do not refuse to consume a food preparation with rocket, bresaola and Grana Padano cheese and have the responsibility (or shared responsibility) of grocery shopping for their household. Consumers who claimed to be allergy sufferers (for rocked or any other ingredient used in the preparations), as well as pregnant and breastfeeding women, were excluded. The subjects were invited to participate in a test session, lasting approximately one hour, at the sensory laboratories of the CREA—Research Centre for Food and Nutrition. They first tasted the two cultivars of rocket (15 g for each cultivar) as a single ingredient and expressed their liking on a nine-point hedonic scale anchored with 1 (“dislike extremely”) and 9 (“like extremely”). Then, they reiterated their evaluation of the cultivars in the recipe. Additionally, they were asked to specify which sensory attribute they liked/disliked for each cultivar and preparation from a list of five terms: hotness, bitter taste, aroma, texture, and leaf shape. Rocket cultivars in both evaluation versions (single ingredient and in the recipe) were presented monadically, coded with three-digit random numbers, and balanced for carry-over effect.

The assessments were carried out in a standard sensory laboratory; participants were seated in isolated sensory booths under standard, controlled conditions (artificial daylight and controlled room temperature at 23 °C). Data were acquired using the FIZZ software 2024 (Biosystèmes, Couternon, France).

Before the evaluations, all consumers were provided with information on the tasting procedures and were asked to sign a written informed consent to participate in the study, which was approved by the Lazio Region “Lazio2” Ethical Committee (Approval n. 0097231/2023). The personal data were anonymously collected and treated in aggregate form, according to the EU general data protection regulation. At the end of the test, consumers were invited to answer questions regarding their rocket’s purchase habits (unpackaged, packaged in a plastic bag, packaged with other salads), consumption habits (as a single ingredient salad, mixed with other types of salad, as an ingredient in a cold dish, e.g., with mozzarella, tomatoes, or as an ingredient in a cooked dish, e.g., strips of meat with rocket) and regarding their preferences of rocket leaves shape. Respondents were rewarded with a voucher after their participation in the study.

### 2.5. Statistical Analyses

Differences between chemical parameters determined in the two cultivars were evaluated for statistical significance by Student’s *t*-test. Panel sensory data were analyzed by two-way analysis of variance (ANOVA), with cultivar and session as main factors (fixed effects), using a 95% confidence interval and a tolerance of 0.0001%. Tukey’s Honest Significant Difference (HSD) test was used for multiple comparisons of sample means at a significance level of *p* ≤ 0.05. On quantitative consumer data (liking), a two-way analysis of variance (ANOVA) was applied, with rocket and preparation (single ingredient or in a recipe) as main factors (fixed effects) and interaction, and consumers (subject) as random effect. Then, an Agglomerative Hierarchical Cluster (AHC) analysis was used to identify clusters based on liking scores; dissimilarity was determined by Euclidean distance, whereas Ward’s Method was used as an agglomeration method. ANOVA and Tukey’s post hoc test were then carried out separately for each cluster.

On categorical consumers’ data, the frequency of use of each sensory attribute for each cultivar and preparation was determined by counting the number of respondents who selected that term as a cause of their liking or disliking. Cochran’s Q test was applied to estimate the significance of differences between each tasted rocket sample for each liked/disliked sensory attribute. For multiple pairwise comparisons between tasted samples, the Critical Difference Sheskin procedure was used as a post hoc test. Finally, to compare the clusters of consumers for frequency of purchase and consumption habits, and rocket leaf shape preference, chi-square was used for testing the independence between rows and columns of the contingency table. Then, each cell of the contingency table was tested against the null hypothesis of independence using Fisher’s exact test. All statistical analyses were performed using XLSTAT 2019.1.2 software (Addinsoft, New York, NY, USA). The significance level was set to *p* < 0.05 in all analyses.

## 3. Results

### 3.1. Sensory Analysis

Results of descriptive sensory analysis of the two rocket cultivars are reported in Table 1. For this study, a list of sensory descriptors similar to that developed in previous studies conducted in our laboratory was used [3]. Ten out of the fourteen sensory attributes showed significant differences, confirming that the two cultivars were markedly different in both appearance, taste, flavour, and texture attributes. As regards appearance Marte had leaves characterized by a more intense and uniform green colour (7.5 vs. 5.7 and 6.7 vs. 5.3 average scores, respectively), the leaf shape was characterized by a more serrated profile (8.8 vs. 7.1), whereas its visual texture appeared more turgid, fleshy, and well hydrated (5.7 vs. 4.9). The odour of the intact leaves revealed a subtle clam-like off-note, which was more pronounced in Denver (1.3 vs. 0.6); conversely, after breaking the leaves by hands, the typical aroma of rocket and cut grass showed the same intensity in both varieties. Marked differences were highlighted in flavour attributes: Marte leaves exhibited higher intensity of both bitterness (6.6 vs. 4.8) and hotness (7.1 vs. 4.7), as evaluated with closed nose, as well as pungency (6.1 vs. 3.6) and rocket flavour (5.1 vs. 4.6), as evaluated after opening the nose during tasting. Texture assessment revealed a thicker leaf consistency during chewing in Marte (5.5 vs. 4.8), along with a slightly higher crispiness, although not statistically significant. The differences in these texture attributes were consistent with the above-mentioned difference in leaves turgidity.

### 3.2. Chemical Determinations

Results from chemical determinations were reported in Table 2. Marte leaves showed a higher level of dry matter, which was reflected in a higher level of soluble solids. The higher level of dry matter and soluble solids in Marte was not due to a higher content of sugars because total sugar content did not differ in the two cultivars, nor significant differences were observed for the two main detected individual sugars, glucose, and fructose.

#### 3.2.1. Glucosinolates

Five major GSLs were identified and quantified, with dimeric 4-mercaptobutyl GSL, (*Rs*)-4-(methylsulfinyl)butyl GSL (glucoraphanin) and 4-(methylsulfanyl)butyl GSL (glucoerucin) representing the three main constituents of the GSL fraction, all three being originated from the methionine biosynthetic pathway. In addition, 4-hydroxyglucobrassicin and glucobrassicin, were also found at a relatively low level. Dimeric 4-mercaptobutyl GSL was, by far, the main GSL in both cultivars, occurring at a markedly higher level (approximately 50% higher) in Marte than in Denver and determining the higher level of total GSLs in the former cultivar. Similarly, glucoraphanin content was higher in Marte, whereas the opposite was observed for glucoerucin.

#### 3.2.2. VOCs

Twenty-four VOCs were identified and quantified in the two rocket cultivars (Table 3), most of which were previously determined by the same analytical approach in different *Diplotaxis tenuifolia* cultivars [3,16]. Most of the quantified VOCs were derived from the metabolism of polyunsaturated fatty acids via the lipoxygenase pathway (C6-aldehydes, alcohols, ketones) or from myrosinase-mediated hydrolysis of GSLs (ITCs, nitriles). For 13 of the determined VOCs, significant differences were observed in the two cultivars, and in 12 out of 13 cases, the level was higher in Denver. As a whole, the content of C6-aldehydes, which represented by far the main fraction of VOCs formed via the lipoxygenase pathway, was not different in the two cultivars, with only a higher level of *E*-2-hexenal in Denver. A higher content in other minor VOCs formed via the lipoxygenase pathway, such as 1-penten-3-one, *Z*-2-Penten-1-ol and 3-Hexen-1-ol, was also observed in Denver. The level of most of the VOCs formed via the hydrolysis of GSLs was higher in Denver, and, in particular, 4-(methylsulfanyl)butyl ITC (erucin), by far the main VOCs detected in the HS-SPME isolate, was markedly higher (nearly double) in Denver than in Marte. A similar trend was also observed for other GSL-derived VOCs, such as 3-butenyl ITC, 4-methylpentyl ITC, and 5-methylsulfanylpentanenitrile, whereas the opposite was observed for 3-(methylsulfanyl)propyl ITC.

### 3.3. Consumers Test

#### 3.3.1. Consumers Sample

The considered sample was composed of male and female proportions of 50%, with an average age of 43.5 ± 13.1 yrs. (age range 18–62 yr.). Respondents’ educational level was 69% lower/upper secondary school and 31% master’s degree. Four percent of the consumer sample declared that they consume rocket once a month, approximately half (48%) of the consumer sample 2–3 times a month, 37% 1–2 times a week, 10% 3–4 times a week and 1% more often.

#### 3.3.2. Hedonic Assessment

Considering the whole consumer group, the overall liking was greater for Denver than for Marte when the product was tasted as a single ingredient, while it did not differ when the two cultivars were evaluated in the recipe (Table 4).

In addition, regardless of rocket cultivar, liking of rocket when consumed in the food preparation was consistently higher than when it was tasted as a single ingredient. This result highlighted the crucial role of the way in which the product is consumed in the judgement of its acceptability. The motivation for the higher overall liking for Denver tasted as a single ingredient lay mainly on the unpleasant sensations related to the higher hotness and bitterness that consumers perceived in Marte, while this difference disappeared when the two cultivars were evaluated in the recipe (Table 5). In fact, 43% and 42% of the respondents selected the hotness and bitterness of Marte leaves as disliked attributes when the rocket was served as a single ingredient, against 14% and 24% for Denver, whereas these frequencies dropped to lower and no more significantly different, counts when both cultivars were assessed in the recipe. However, results collected on the overall sample of consumers did not reflect the preferences of all consumers. Five clusters of consumers were identified from agglomerative hierarchical clustering (AHC) analysis on overall liking data. CL1 (29%), one of the largest clusters, was characterized by relatively high liking scores, except for Marte as an individual ingredient (Table 4): clearly, for this group of consumers, the intense hotness, bitterness, and aroma of Marte were objectionable attributes that motivated the markedly lower liking when tasting this cultivar as a single ingredient, whereas these attributes lost their unpleasant character in the recipe (Table S1). Consumers of the largest cluster, CL2 (30%), appreciated all kinds of rocket and ways of consumption, even though liking scores tended to increase for food preparation (Table 4). These people did not find offensive the stronger flavour of Marte, and this was so true that, while they attributed the lowest liking score to Denver tasted as a single ingredient, the highest preference was assigned to Marte as tasted in the recipe. On the contrary, CL3 (15%) did not like both cultivars when evaluated as a single ingredient, particularly the cultivar Marte, whereas they released positive evaluations for both cultivars when evaluated in the recipe (Table 4). Again, the main driver of the low overall liking of rocket leaves tasted as a single ingredient lay in their bitter taste, intense aroma, and hotness (this latter only for Marte) (Table S1). The CL4 (16%) showed, in general, high liking scores but, interestingly, expressed a higher liking for Marte than for Denver in the single ingredient tasting mode (Table 4). These people tended to appreciate the hotness and aroma of Marte and not to report disliked attributes (Table S1). The smaller cluster, CL5 (10%) instead, was characterized by a relatively low liking for all rocket samples and preferred Denver to Marte, regardless of the condition of tasting. In particular, they disliked Marte when tasted as a single ingredient (Table 4). Again, the reason for this dislike lay in the hotness and bitter taste of Marte leaves, as highlighted by the high frequencies that are reported as dislike attributes (Table S1).

#### 3.3.3. Purchase and Consumption Habits, Preferences for Rocket Leaves Shape

The vast majority of the consumers sample (86%) typically purchase rockets as a packaged product, while the purchase of rockets packaged in salad mixes or unpackaged is much less frequent (Table 6).

As regards consumption habits, the most common way is as an ingredient in a cold dish (83% of the respondents), mixed with other salads (66%) and as an ingredient in a cooked dish (62%), whereas consumption as a single ingredient salad (39%) is by far the less usual condition of consumption (Table 6). Finally, answers to the questionnaire showed a clear positive attitude of consumers toward serrated-shaped leaves with respect to round-shaped leaves (66% vs. 34%). A few differences in these habits and preferences were observed between the consumers’ clusters (Table 6). The CL4 showed a more pronounced tendency to consume rocket as a single-ingredient salad, to purchase it unpackaged, and at the same time showed a marked preference for the serrated-shaped rocket leaves. On the contrary, CL3 showed a significantly lower attitude to consuming a rocket as a single ingredient and a higher preference for a round-shaped rocket. Finally, CL5 was characterized by the lowest global number of answers expressed about the usual consumption habits, while they did not show any preference (50% for each leaf shape) about the type of leaf shape.

## 4. Discussion

### 4.1. Chemical and Sensory Characteristics

The approach followed in the present study to investigate consumers’ preferences for rocket leaves was based on the use of only two cultivars but paying attention to select them in such a way that they largely differed in key sensory and, in particular, flavour attributes. This could make clearer the identification of the sensory attributes that drive the consumers’ preferences. In one previous study, where seven accessions of *Eruca sativa* were evaluated, the limited variability in some of the key sensory attributes, such as bitterness, among the analysed rocket types [5] may have brought to underestimate their contribution to the global liking of the rocket by consumers. As expected, results of sensory analyses confirmed that Marte was characterized by a definitely stronger flavour than Denver, with much more intense sensations of bitterness, hotness and pungency as perceived by the sensory panellists. Interestingly, these three attributes covaried in the two cultivars, and, in particular, hotness and bitterness were linked to each other. In previous investigations, while these two attributes appeared to be associated in a study on commercial samples of *Diplotaxis tenuifolia* [2], they did not covary in a set of 7 accessions of *Eruca sativa* [5]. The higher bitterness in Marte corresponded to the higher level of GSLs detected in this cultivar, which was due to the higher content of the dimeric 4-mercaptobutyl GSL. While it is generally accepted that in *Brassicaceae* species, bitterness is associated with the presence of GSLs and ITCs, for some species, including rockets, there is little robust evidence relating this attribute to individual GSL or ITC compounds [21]. This is mainly due to the lack of available food grade standards for some key GSLs and ITCs and the resulting lack of information on their taste threshold. As regards rocket, Pasini et al. [7] observed an association between bitterness intensity and dimeric 4-mercaptobutyl GSL in a selection of 37 accessions of *Eruca sativa* and *Diplotaxis tenuifolia,* consistently with the results of the present study, whereas this relationship was not confirmed in other investigations [2,5]. While the absence of a contribution of glucoraphanin to bitterness has been repeatedly reported, and the same has also been suggested for glucoerucin [21]; still, it remains to elucidate the role of the dimeric 4-mercaptobutyl GSL and future isolation of this compound from rocket leaves and sensory evaluation of the purified compound could give an answer to this question. In the present study, the content of sugars was also determined because previous investigations suggested their role in partially masking the bitter perception produced by GSLs, thus resulting in a reduced intensity of bitterness perception [2,4]. However, the level of sugars in the two cultivars did not differ significantly, so it was unlikely that they may contribute to differences in bitterness perception. As regards pungency, it is known that this sensation is due to volatile compounds because it may be sensed via the orthonasal route after crushing some leaves close to the nose due to the stimulation of the trigeminal nerve branches in the nasal cavity. Thus, if it is assumed that bitterness is mainly due to GSLs, which are non-volatile compounds, pungency will be produced by separate compounds. In addition, to further stress the distinct origin of the two sensations, it should be remembered that receptors of bitterness, which are taste receptors, are separate from receptors of pungency, which are transient receptor potential (TPR) ion channels, involved in the perception of chemestethic sensations. Pasini et al. [7] observed an association between pungency and total GSL content, consistent with the results of the present study, and this would suggest that pungency might be produced by VOCs formed via the hydrolysis of GSLs. However, in the present study, as in earlier investigations, it was not possible to consistently relate any of the determined VOCs to the intensity of pungency. Based on previous observations [16,22], it is plausible that the perception of pungency may be due to the formation of very unstable and short-lived GSL hydrolysis products that the isolation procedure used in the present study was not able to capture. Other spectrometric techniques, such as Proton Transfer Reaction (PTR) methods, which may allow online detection of volatile compounds could be used in future to further investigate the formation of unstable transient compounds responsible for pungency. In any case, in the present study, the limitations of the isolation procedure and of the GC-MS method used [16] did not allow for the detection of the hydrolysis products of the main constituent of the GSL fraction, i.e., the dimeric 4-mercaptobutyl GSL. Hotness, similar to pungency, is mediated by transient receptor potential (TPR) ion channels but, in principle, may be produced by both volatile and non-volatile compounds. Moreover, being a long-lasting sensation, it is characterized by very different temporal properties when compared to pungency. Results from this study suggested a relationship between total GSL content and hotness, similar to what was observed for pungency. Moreover, analysis of VOCs also showed that the higher content of glucoerucin found in Denver corresponded to the higher level of its hydrolysis product erucin, which has been previously identified as one of the key odorants of rocket and described with a radish-like odour note [16]. However, the difference in erucin level seemed not to have a great impact on the perceived typical rocket aroma and flavour. In addition, the levels of the C6-aldehydes (hexanal, 2- and 3-hexenals), previously recognized as key odorants contributing to the green olfactory notes of rocket leaves [16], did not differ in the two cultivars, and correspondingly no significant differences were perceived in cut grass odour and fresh green flavour. Finally, the subtly perceived rotten leaves and clam-like off-odours have been reported to be due to the presence of sulphur (rotten leaves) and still unknown (clam-like) compounds that could be better detected by using a specific isolation method, as described earlier [23]; however, in this study, dealing with fresh rocket samples, this was not considered as a priority of the VOCs’ characterization, and so a different isolation procedure, mainly focused on rocket aroma VOCs, was selected.

### 4.2. Consumers’ Preferences

A previous investigation aimed at exploring the relationships between sensory attributes of mixed salad leaves and liking among Italian consumers highlighted that both liking and perception of freshness were related to appearance attributes, such as green colour, salad assortments and leaf turgidity [24]. In that study, mixed salads formed by five different species were considered, whereas in the present study, the focus was on the sole rocket species. Considering the very distinctive and prominent flavour of the rocket, one might expect, in this case, a more important role of flavour with respect to appearance and texture. Consequently, as explained above, the selection of the two sample cultivars was guided by the search for the largest possible difference in flavour. To strengthen the reliability of the results of the consumer test, a relatively large amount of rocket leaves, 15 g for the single ingredient and 3 g for the recipe was provided to the consumers to limit the confounding effect of biological variability between different leaves. In addition, by number (100 volunteers), recruitment criteria (in particular, being habitual consumers of rocket and accept the recipe of rocket, bresaola and Grana Padano cheese) and demographic profile, the results of the consumer study are not intended to be exploratory but can be extended to a wider population of Italian consumers, although the recruitment for logistical reasons has been limited to the city of Rome and its surroundings. The obtained results confirmed that, even though a subset of consumers may prefer rocket cultivars with increased hotness, pungency and bitterness, most consumers generally do not like a high intensity of these attributes and prefer milder rocket types when used as a single-ingredient salad. On this point, the preferences expressed by Italian consumers in the present study appeared to be in line with those previously observed among UK consumers in previous studies [2,4]. In addition, in the present study, both bitterness and hotness seemed to act as main drivers of rocket liking/disliking, confirming the findings of a previous study on a large set of *Diplotaxis tenuifolia* rocket samples [2] and disconfirming previous observations on *Eruca sativa* accessions, where hotness and not bitterness appeared to drive rocket liking/disliking [4]. But, as remarked above, this latter finding may be due to small differences in bitterness between the accessions tested. Moreover, the main finding of the present study was that the differences in liking expressed when tasting a rocket as a single ingredient disappeared when the rocket was evaluated in a recipe. This was observed both in the sample of consumers considered as a whole and in the five clusters identified within it. Indeed, in the largest cluster (CL2), the preference expressed for the milder cultivar when tasted as a single ingredient tended to be reversed when evaluating the rocket in the recipe. Thus, what was judged to be an undesirable attribute, such as an intense bitterness and hotness, when tasting the rocket as a single ingredient, turned out to be a desirable, or an insignificant, trait when the rocket was tasted within a recipe with other ingredients. This means that drawing general conclusions about consumers’ preferences for different rocket cultivars based solely on tests conducted by tasting it as a single ingredient could be risky and represent an oversimplification. Rocket, from a flavour standpoint, is a particular species and not comparable to other leafy salad greens, such as lettuce, endive, chicory, watercress, and others. This is because it is characterized by a very intense flavour, with pronounced attributes of hotness, bitterness, and pungency, which make rocket not commonly consumed as a standalone ingredient. As a result, in this case, as highlighted in the present study, more complex and elaborate approaches may be needed in the evaluation of consumer preferences.

From data collected by the questionnaire, it clearly emerged that, in accordance with what is assumed by the producers, Italian consumers clearly prefer rocket types characterized by a serrated-shaped leaf, with respect to a round-shaped leaf. However, appearance attributes seemed to play a secondary role in determining the global liking of rockets compared to flavour attributes when analyzing the frequency of answers of liked/dislikes attributes from consumers. This result also confirmed previous observations on UK consumers [2]. Moreover, according to consumer evaluations, texture seemed to play a minor role in determining the differences in liking between the two cultivars.

Analysis of the results of the consumer test also allowed us to identify five clusters of consumers. Interestingly, comparative analysis of the data obtained by the tasting experiment and data collected by the questionnaire suggested the occurrence of some associations between preferences and purchase/consumption habits useful for a more informative characterisation of some subsets of consumers. For example, the CL4 (16% of the consumer sample) was composed of people who liked rockets with an intense flavour, high bitterness and hotness, and this corresponded to habits of purchase (toward unpackaged), of consumption (toward consumption as a single ingredient salad) and preference of leaf shape (for the serrated shape), so that all these observations suggested a coherent profile of these consumers that can be described as “lovers of typical, strong, wild rocket”. These people seemed to prefer a kind of rocket that is characterized by a stronger flavour, which in turn may be associated with an image of a product that is more natural and subjected to fewer industrial treatments (processing, packaging, etc.). As another example, CL3 (15% of the consumers) expressed low liking scores for rockets when tasted as a single ingredient, seemed to dislike high bitterness and hotness, and coherently showed consumption habits (lower attitude to consuming rocket as a single ingredient) and preference for leaf shape (higher for round-shaped leaf) that pointed to a consumers’ profile that could be described as “lovers of mild flavour, round-shaped rocket”. Both the liking test (on average, low liking scores) and the questionnaire data (a relatively low number of expressed answers on usual consumption habits, no preference for leaf shape) suggested that people in CL5 (10% of consumers) could be described as “little attracted by rocket”. Finally, the larger clusters, CL1 and CL2, seemed to represent the “mainstream profile” of rocket consumers, both in terms of liking and habits. This group of people liked rockets better if in a recipe instead of as a single ingredient, preferred milder cultivars when tasting rockets as a standalone ingredient, and generally purchased packaged rockets.

All this information allows us to envision the possibility of implementing product differentiation strategies aimed at commercially exploiting those rocket cultivars with specific sensory properties that could meet the expectations of particular consumer groups. For example, cultivars with a strong flavour could be the focus of marketing activities targeting consumers we have described above as “lovers of the typical, strong, wild rocket”. Moreover, on package information, suggestions for more appropriate culinary use could be provided based on the different sensory profiles of various cultivars. For example, for cultivars with a strong flavour, their use in recipes could be recommended, while for cultivars with a milder flavour, consumption as a single-ingredient salad could be suggested.

## 5. Conclusions

In previous investigations on rocket quality, it has been emphasized that efforts to implement breeding and marketing strategies should be based on knowledge of the reasons for consumers liking or disliking of rockets and on quantification of their sensory traits [4,25]. Results from the present study did not show differences in wild rocket preferences between Italian and UK consumers and highlighted that the peculiar sensory characteristics of rockets require the adoption of more complex and sophisticated experimental approaches for the evaluation of consumer preferences. Assessing preferences via tasting leaves exclusively as a single ingredient is a suitable approach for the evaluation of leafy salads other than rockets. However, this approach could lead to incomplete or misleading indications in the case of rocket leaves, where it is crucial to take into account the influence of the specific ways by which the rocket is actually consumed. From this perspective, it might be interesting in the future to evaluate the effect on consumer liking of other ways of consuming rockets, such as in a mixed salad or in cooked dishes. Finally, the indications gathered on consumer preferences suggested possible strategies for product differentiation and for the promotion of specific cultivars based on their peculiar sensory traits.

## Figures and Tables

**Table 1:** Sensory profile of the two wild rocket cultivars, Denver and Marte, as determined by sensory descriptive analysis: mean values, *p* values from a 2-way ANOVA (with cultivar and session as factors) and results from multi-comparison Tukey’s test for each sensory attribute.

Sensory Attribute	Rocket Cultivar		ANOVA *p*		
	Denver	Marte	Cultivar	Session	Cultivar × Session
*Evaluation of the intact leaves*					
Colour intensity	5.7 b ^1^	7.5 a	<0.001	0.616	0.963
Colour uniformity	5.3 b	6.7 a	<0.001	0.236	0.377
Leaf shape	7.1 b	8.8 a	<0.001	0.515	0.789
Integrity (of leaves)	7.0 a	7.5 a	0.095	0.385	0.979
Turgidity (of leaves)	4.9 b	5.7 a	0.011	0.421	0.297
Clam-like off odour	1.3 a	0.6 b	0.048	0.655	0.865
Rotten leaves off-odour	0.4 a	0.1 a	0.095	0.267	0.419
*Evaluation after breaking the leaves by hands*					
Typical rocket odour	6.3 a	6.5 a	0.577	0.515	0.475
Cut grass odour	3.8 a	4.3 a	0.350	0.711	0.780
*Evaluation after tasting*					
Bitter taste	4.8 b	6.6 a	<0.001	0.805	0.071
Hotness	4.5 b	7.1 a	<0.001	0.189	0.988
Pungency	3.6 b	6.1 a	<0.001	0.838	0.641
Typical rocket flavour	6.3 b	7.4 a	<0.001	0.221	0.531
Fresh green flavour	4.6 a	5.1 a	0.123	0.190	0.711
Rotten leaves off-flavour	0.4 a	0.2 a	0.454	0.284	0.848
Crispness	5.0 a	5.6 a	0.114	0.106	0.917
Chewing consistency	4.8 b	5.5 a	0.042	0.550	0.683
Juiciness	4.0 a	3.8 a	0.627	0.783	0.602

^1^ Mean values within a row with different letters differ (*p* < 0.05) for Tukey’s post hoc test.

**Table 2:** Dry matter, soluble solids, sugar and glucosinolate content of the two wild rocket cultivars, Denver and Marte: mean value, standard deviation, and *p* value from Student’s *t*-test.

Parameter	Rocket Cultivar		Student’s *t*-Test *p*
	Denver	Marte	
Dry matter (%)	7.85 (0.03)	8.83 (0.08)	<0.001
Soluble solids (° Brix)	5.4 (0.1)	6.0 (0.2)	0.010
*Sugars (mg/g f.w.)*			
Fructose	0.31 (0.05)	0.43 (0.09)	0.118
Glucose	2.38 (0.40)	2.19 (0.46)	0.630
Total sugars	2.69 (0.46)	2.63 (0.56)	0.882
*Glucosinolates (mg/100 g f.w.)*			
Glucoraphanin	2.52 (0.29)	3.11 (0.07)	0.007
Glucoerucin	0.93 (0.11)	0.54 (0.05)	<0.001
Dimeric 4-mercaptobutyl glucosinolate	11.84 (0.55)	17.58 (0.47)	<0.001
4-Hydroxyglucobrassicin	0.09 (0.02)	0.06 (0.00)	0.019
Glucobrassicin	0.02 (0.02)	0.04 (0.04)	0.447
Total glucosinolates	15.39 (0.78)	21.32 (0.56)	<0.001

**Table 3:** Levels of VOCs obtained by semi-quantitative determination (expressed as the equivalent concentration of an internal standard, as mg kg^−1^ f.w.) on crushed rocket leaves of the two wild rocket cultivars: mean value, standard deviation, and *p* value from Student’s *t*-test.

Compound	Rocket Cultivar		Student’s *t*-Test *p*
	Denver	Marte	
2-Ethylfuran	0.042 (0.010)	0.034 (0.003)	0.266
1-Penten-3-one	0.020 (0.001)	0.016 (0.000)	0.002
Hexanal	0.021 (0.001)	0.019 (0.002)	0.208
Tetrahydrothiophene	0.18 (0.04)	0.11 (0.01)	0.030
E-3-Hexenal	0.28 (0.03)	0.24 (0.02)	0.084
Z-3-Hexenal	2.10 (0.14)	2.00 (0.20)	0.503
Z-2-Hexenal	0.34 (0.02)	0.31 (0.03)	0.192
E-2-Hexenal	0.47 (0.03)	0.39 (0.04)	0.042
2-Octanone	0.056 (0.003)	0.053 (0.001)	0.118
Z-2-Penten-1-ol	0.014 (0.000)	0.013 (0.000)	<0.001
6-Methyl-5-hepten-2-one	0.003 (0.000)	0.008 (0.004)	0.088
5-Methyl-hexanenitrile	0.013 (0.002)	0.008 (0.000)	0.008
3-Hexen-1-ol	0.17 (0.00)	0.13 (0.00)	<0.001
Nonanal	0.003 (0.000)	0.004 (0.001)	0.100
2,4-hexadienal	0.10 (0.02)	0.07 (0.00)	0.060
2,4-hexadienal	0.23 (0.04)	0.16 (0.01)	0.046
3-Butenyl isothiocyanate	0.63 (0.04)	0.39 (0.06)	0.004
2,4-heptadienal	0.008 (0.001)	0.010 (0.002)	0.177
Camphor	0.104 (0.000)	0.099 (0.001)	0.001
4-Methylpentyl isothiocyanate	0.17 (0.02)	0.10 (0.01)	0.003
5-Methylsulfanylpentanenitrile	0.21 (0.00)	0.11 (0.01)	<0.001
3-(Methylsulfanyl)propyl isothiocyanate	0.62 (0.00)	0.79 (0.06)	0.008
4-(Methylsulfanyl)butyl isothiocyanate (erucin)	23.50 (0.18)	12.28 (0.67)	<0.001
Nonanoic acid	0.003 (0.002)	0.003 (0.000)	0.784
Sum of C6 aldehydes	3.20 (0.21)	2.93 (0.28)	0.254
Sum of glucosinolate hydrolysis products	25.15 (0.18)	13.68 (0.69)	<0.001

**Table 4:** Overall liking of the two rocket cultivars, tasted as a single ingredient and as a part of a recipe, for the overall sample and for the clusters of consumers obtained from agglomerative hierarchical clustering. Analysis of variance (ANOVA) and Tukey’s honest significant difference (HSD) test.

	Rocket Cultivar		Overall (n = 100)	CL 1 (n = 29)	CL 2 (n = 30)	CL 3 (n = 15)	CL 4 (n = 16)	CL 5 (n = 10)
Single ingredient	Denver		6.7 b ^1^	7.4 a	7.7 c	4.5 b	5.6 b	6.6 a
Single ingredient	Marte		5.5 c	4.9 b	7.2 bc	3.1 c	7.4 a	2.7 c
Recipe	Denver		7.5 a	7.3 a	7.9 ab	7.7 a	7.8 a	5.8 ab
Recipe	Marte		7.3 a	7.6 a	8.5 a	6.9 a	7.0 a	4.4 bc
	Sample	F	28.817	47.959	10.342	31.447	13.394	11.915
ANOVA		*p*	<0.001	<0.001	<0.001	<0.001	<0.001	<0.001
			**F**	***p***				
	Subject		2.922	<0.001				
	Preparation		23.633	<0.001				
	Rocket		89.620	<0.001				
	Preparation × Rocket		14.729	<0.001				

^1^ Mean values within a column with different letters differ (*p* < 0.05) for Tukey’s post hoc test.

**Table 5:** Frequencies of liked/disliked attributes (number of respondents who selected those terms as a cause of their liking or disliking) as specified by consumers (n = 100).

	Single Ingredient		Recipe		Cochran’s Q (*p*) ^1^
	Denver	Marte	Denver	Marte	
*Liked attributes*					
Hotness	38 b ^2^	35 b	15 a	24 ab	<0.001
Bitter taste	36 ab	21 a	27 ab	39 b	0.007
Aroma	40 a	26 a	72 b	59 b	<0.001
Texture	61	65	73	73	0.083
Leaf shape	38 b	48 b	0 a	0 a	<0.001
*Disliked attributes*					
Hotness	14 a	43 b	5 a	9 a	<0.001
Bitter taste	25 b	42 c	8 a	11 ab	<0001
Aroma	14 ab	24 b	4 a	12 a	<0.001
Texture	11	9	11	12	0.920
Leaf shape	13 b	9 b	0 a	0 a	<0.001

^1^ Significance of differences in the frequency scores between each tasted rocket sample for each liked/disliked sensory attribute according to Cochran’s Q test (*p* < 0.05). ^2^ Different letters in a row denoted significant differences in the frequency scores according to multiple pairwise comparisons calculated by using the critical difference Sheskin procedure.

**Table 6:** Frequencies (number of respondents), percentage frequencies of purchase and consumption habits, and rocket leaf shape preferences for the overall consumer sample and for the clusters of consumers obtained from agglomerative hierarchical clustering.

	All (n = 100)	Cluster 1 (n = 29)		Cluster 2 (n = 30)		Cluster 3 (n = 15)		Cluster 4 (n = 16)		Cluster 5 (n = 10)		*χ*^2^ Test (*p*) ^1^
	n	n	%	n	%	n	%	n	%	n	%	
**Usual purchase**												
Unpackaged	31	10	34.5	8	26.7 * ^2^	5	33.3	6	37.5	3	30	
Packaged	86	26	89.7	27	90.0	12	80.0	14	87.5	7	70	
Packaged with other salads	28	8	27.6	11	36.7	5	33.3	3	18.8 *	1	10 *	0.039
**Usual consumption**												
Single ingredient salad	39	12	41.4	11	36.7	4	26.7 *	8	50.0 ^§ 3^	4	40	
Mixed with other salads	66	19	65.5	23	76.7	10	66.7	8	50.0	6	60	
As an ingredient in a cold dish	83	26	89.7	24	80.0	13	86.7	12	75.0	8	80	
As an ingredient in a cooked dish	62	22	75.9	20	66.7	11	73.3 ^§^	8	50.0	2	20 *	<0.001
**Preference for**												
Round-shaped leaf	34	8	27.6 *	11	36.7	8	53.3 ^§^	2	12.5 *	5	50 ^§^	
Serrated-shaped leaf	66	21	72.4	19	63.3	7	46.7 *	14	87.5 ^§^	5	50 *	<0.001

^1^ The chi-square (*χ*^2^) test was used for testing the independence between rows and columns of the contingency table formed by the percentage frequency values (*p* < 0.05). ^2^ The symbol * denoted that the value was significantly lower than the others in the same row according to the Fisher exact test (for α = 0.05). ^3^ The symbol ^§^ denoted that the value was significantly higher than the others in the same row according to the Fisher exact test (for α = 0.05).

## Data Availability

The original contributions presented in the study are included in the article/Supplementary Materials, further inquiries can be directed to the corresponding author.

## Supplementary Materials

The following supporting information can be downloaded at https://www.mdpi.com/article/10.3390/foods13111699/s1. Table S1: Frequencies of liked/disliked attributes (number of respondents who selected those terms as a cause of their liking or disliking) as specified by consumers’ clusters.
