# Supplementary material for: Sensory Attributes Driving Preference for Wild Rocket (Diplotaxis tenuifolia) Leaves Tasted as a Single Ingredient and as a Part of a Recipe
Source: Foods. 2024 May 28;13(11):1699. doi: 10.3390/foods13111699 (PMC11172029; doi:10.3390/foods13111699)
Supplement: Supplementary file 1 [file foods-13-01699-s001.zip › foods-3018551-supplementary.pdf]

Table S1. Frequencies of liked/disliked attributes (number of respondents who selected those terms as a cause of their liking or disliking) as specified by consumers' clusters.

CLUSTER 1 (n=29)

Liked Attributes	Denver (single ingredient)		Marte (single ingredient)		Denver (recipe)		Marte (recipe)		Cochran's Q
	n	%	*n*	%	*n*	%	*n*	%	(*p*)[1]
Hotness	*13*	44.8 b[2]	*5*	17.2 ab	*4*	13.8 a	*8*	27.6 ab	0.017
Bitter taste	*11*	37.9 ab	*5*	17.2 a	*7*	24.1 ab	*14*	48.3 b	0.009
Aroma	*14*	48.3 b	*4*	13.8 a	*19*	65.5 b	*17*	58.6 b	< 0.001
Texture	*17*	58.6	*9*	65.5	*22*	75.9	*21*	72.4	0.306
Leaf shape	*11*	37.9 b	*15*	51.7 b	*0*	0 a	*0*	0 a	< 0.001

Disliked Attributes	Denver (single ingredient)		Marte (single ingredient)		Denver (recipe)		Marte (recipe)		Cochran's Q
	n	%	*n*	%	*n*	%	*n*	%	(*p*)
Hotness	*5*	17.2 a	*15*	51.7 b	*1*	3.4 a	*2*	6.9 a	< 0.001
Bitter taste	*5*	17.2 a	*16*	55.2 b	*3*	10.3 a	*2*	6.9 a	< 0.001
Aroma	*0*	0.0 a	*13*	44.8 b	*1*	3.4 a	*3*	10.3 a	< 0.001
Texture	*0*	0.0	*1*	3.4	*2*	6.9	*5*	17.2	0.054
Leaf shape	*4*	13.8	*3*	10.3	*0*	0.0	*0*	0.0	0.045

Notes.[1] Significance of differences of the frequency scores between each tasted rocket samples for each liked/disliked sensory attribute according to Cochran's Q test ($p<0.05$).
[2] Different letters in a row denoted significant differences of the frequency scores according to multiple pairwise comparisons calculated by using the critical difference Sheskin procedure.

CLUSTER 2 (n=30)

Liked Attributes	Denver (single ingredient)		Marte (single ingredient)		Denver (recipe)		Marte (recipe)		Cochran's Q
	n	%	*n*	%	*n*	%	*n*	%	(*p*)[1]
Hotness	*11*	36.7 ab[2]	*15*	50.0 b	*3*	10.0 a	*6*	20.0 a	0.002
Bitter taste	*14*	46.7	*8*	26.7	*7*	23.3	*15*	50.0	0.022
Aroma	*15*	50.0 ab	*11*	36.7 a	*25*	83.3 c	*20*	66.7 bc	0.001
Texture	*19*	63.3	*22*	73.3	*22*	73.3	*25*	83.3	0.308
Leaf shape	*12*	40.0 b	*14*	46.7 b	*0*	0.0 a	*0*	0.0 a	< 0.001

Disliked Attributes	Denver (single ingredient)		Marte (single ingredient)		Denver (recipe)		Marte (recipe)		Cochran's Q
	n	%	*n*	%	*n*	%	*n*	%	(*p*)
Hotness	*3*	10.0 a	*11*	36.7 b	*3*	10.0 a	*1*	3.3 a	0.002
Bitter taste	*4*	13.3	*4*	13.3	*3*	10.0	*1*	3.3	0.463
Aroma	*2*	6.7	*1*	3.3	*0*	0.0	*0*	0.0	0.300
Texture	*5*	16.7	*3*	10.0	*6*	20.0	*2*	6.7	0.414
Leaf shape	*5*	16.7 b	*2*	6.7 ab	*0*	0.0 a	*0*	0.0 a	0.014

Notes.[1] Significance of differences of the frequency scores between each tasted rocket samples for each liked/disliked sensory attribute according to Cochran's Q test ($p<0.05$).
[2] Different letters in a row denoted significant differences of the frequency scores according to multiple pairwise comparisons calculated by using the critical difference Sheskin procedure.

CLUSTER 3 (n=15)

Liked Attributes	Denver (single ingredient)		Marte (single ingredient)		Denver (recipe)		Marte (recipe)		Cochran's Q
	n	%	*n*	%	*n*	%	*n*	%	(*p*)[1]
Hotness	*4*	26.7	*3*	20.0	*5*	33.3	*5*	33.3	0.786
Bitter taste	*4*	26.7	*1*	6.7	*5*	33.3	*6*	40.0	0.154
Aroma	*3*	20.0 a[2]	*3*	20.0 a	*11*	73.3 b	*11*	66.7 ab	0.002
Texture	*10*	66.7	*9*	60.0	*11*	73.3	*8*	53.3	0.543
Leaf shape	*5*	33.3 ab	*9*	60.0 b	*0*	0.0 a	*0*	0.0 a	< 0.001

Disliked Attributes	Denver (single ingredient)		Marte (single ingredient)		Denver (recipe)		Marte (recipe)		Cochran's Q
	n	%	*n*	%	*n*	%	*n*	%	(*p*)
Hotness	*1*	6.7 a	*9*	60.0 b	*0*	0.0 a	*1*	6.7 a	< 0.001
Bitter taste	*10*	66.7 b	*12*	80.0 b	*1*	6.7 a	*5*	33.3 ab	< 0.001
Aroma	*7*	46.7 b	*6*	40.0 ab	*1*	6.7 a	*3*	20.0 ab	0.024
Texture	*2*	13.3	*1*	6.7	*2*	13.3	*3*	20.0	0.753
Leaf shape	*1*	6.7	*1*	6.7	*0*	0.0	*0*	0.0	0.392

Notes.[1] Significance of differences of the frequency scores between each tasted rocket samples for each liked/disliked sensory attribute according to Cochran's Q test ($p<0.05$).
[2] Different letters in a row denoted significant differences of the frequency scores according to multiple pairwise comparisons calculated by using the critical difference Sheskin procedure.

CLUSTER 4 (n=16)

Liked Attributes	Denver (single ingredient)		Marte (single ingredient)		Denver (recipe)		Marte (recipe)		Cochran's Q
	n	%	*n*	%	*n*	%	*n*	%	(*p*)[1]
Hotness	*4*	25.0 ab[2]	*10*	62.5 b	*2*	12.5 a	*4*	25.0 ab	0.010
Bitter taste	*3*	18.8	*5*	31.3	*4*	25.0	*3*	18.8	0.801
Aroma	*5*	31.3 a	*8*	50.0 ab	*13*	81.3 b	*8*	50.0 ab	0.041
Texture	*7*	43.8	*8*	50.0	*11*	68.8	*12*	75.0	0.165
Leaf shape	*6*	37.5 b	*5*	31.3 ab	*0*	0.0 a	*0*	0.0 a	0.005

Disliked Attributes	Denver (single ingredient)		Marte (single ingredient)		Denver (recipe)		Marte (recipe)		Cochran's Q
	n	%	*n*	%	*n*	%	*n*	%	(*p*)
Hotness	2	12.5	1	6.3	0	0.0	2	12.5	0.468
Bitter taste	6	37.5 b	3	18.8 ab	0	0.0 a	1	6.3 ab	0.015
Aroma	3	18.8	1	6.3	0	0.0	2	12.5	0.290
Texture	3	18.8	2	12.5	0	0.0	0	0.0	0.145
Leaf shape	2	12.5	2	12.5	0	0.0	0	0.0	0.261

Notes.[1] Significance of differences of the frequency scores between each tasted rocket samples for each liked/disliked sensory attribute according to Cochran's Q test ($p<0.05$).
[2] Different letters in a row denoted significant differences of the frequency scores according to multiple pairwise comparisons calculated by using the critical difference Sheskin procedure.

CLUSTER 5 (n=10)

Liked Attributes	Denver (single ingredient)		Marte (single ingredient)		Denver (recipe)		Marte (recipe)		Cochran's Q
	n	%	*n*	%	*n*	%	*n*	%	(*p*)[1]
Hotness	*6*	60.0	2	20.0	*1*	10.0	*1*	10.0	0.026
Bitter taste	*4*	40.0	2	20.0	*4*	40.0	*1*	10.0	0.356
Aroma	*3*	30.0	0	0.0	*4*	40.0	*4*	40.0	0.105
Texture	*8*	80.0	7	70.0	*7*	70.0	*7*	70.0	0.912
Leaf shape	*4*	40.0 ab[2]	5	50.0 b	*0*	0.0 a	*0*	0.0 a	0.004

Disliked Attributes	Denver (single ingredient)		Marte (single ingredient)		Denver (recipe)		Marte (recipe)		Cochran's Q
	n	%	*n*	%	*n*	%	*n*	%	(*p*)
Hotness	*3*	30.0 ab	*7*	70.0 b	*1*	10.0 a	*3*	30.0 ab	0.016
Bitter taste	*0*	0.0 a	*7*	70.0 b	*1*	10.0 a	*2*	20.0 ab	0.002
Aroma	*2*	20.0	*3*	30.0	*2*	20.0	*4*	40.0	0.724
Texture	*1*	10.0	*2*	20.0	*1*	10.0	*2*	20.0	0.861
Leaf shape	*1*	10.0	*1*	10.0	*0*	0.0	*0*	0.0	0.572

Notes.[1] Significance of differences of the frequency scores between each tasted rocket samples for each liked/disliked sensory attribute according to Cochran's Q test ($p<0.05$).
[2] Different letters in a row denoted significant differences of the frequency scores according to multiple pairwise comparisons calculated by using the critical difference Sheskin procedure.
